# Supplementary material for: Mapping the functional impact of non-coding regulatory elements in primary T cells through single-cell CRISPR screens
Source: Genome Biol. 2024 Feb 2;25:42. doi: 10.1186/s13059-024-03176-z (PMC10835965; doi:10.1186/s13059-024-03176-z)
Supplement: Supplementary file 1 — Additional file 1: Fig. S1. A) Schematic of dCas9-KRAB lentiviral constructs tested. B) Bar plot showing the percentage of cells retaining protein expression for different target genes (CD4, CD81, BST2, ATP1B3) 10 days after TSS-targeting gRNA transduction into primary CD4+ T cells expressing different dCas9-repressor constructs, analysed by flow cytometry and normalised to the corresponding non-targeting gRNA control sample. gRNA #1 and #2 refer to two different gRNA designs for a given TSS. C) Bar plots for the same experiment as B), showing flow cytometry data for days 6, 10 and 16 post-gRNA transduction. D) Normalised expression levels of ATP1B3, measured by 10X Genomics 3’ scRNA-seq 11 days after the corresponding targeting (red) or non-targeting (grey) gRNAs were transduced into primary CD4+ T cells expressing a CBh-ZIM3-dCas9 repressor construct. Dashed line indicates median expression level in cells with non-targeting controls. E) Percentage of cells showing protein downregulation of the target gene after CRISPRi by flow cytometry (x-axis) versus significance (-log10 FDR) of downregulation of the target gene (mRNA) by 3’ scRNA-seq analysis (y-axis), normalised to the corresponding NT control. Pearson r2 = 0.98. Fig. S2. A) Distributions of the total UMIs per cell, number of detected genes per cell, and fraction of reads mapping to mitochondrial genes, used for quality control of the scRNA-seq data. Each violin corresponds to a technical replicate (channel in a 10X chip); colours indicate different 10X chips. The dotted lines indicate the thresholds used for each replicate to exclude poor-quality cells. B) Same as Fig. 2C but split per technical replicate. C) Barplot showing the number of cells where the same gRNA is the most abundant in both the cDNA and gRNA libraries (concordant, blue); the most abundant gRNA is different between libraries (discordant, orange); or no gRNA information was recovered from the cDNA library (yellow) D) Scatter plot of the relat [file 13059_2024_3176_MOESM1_ESM.pdf]

Figure S1

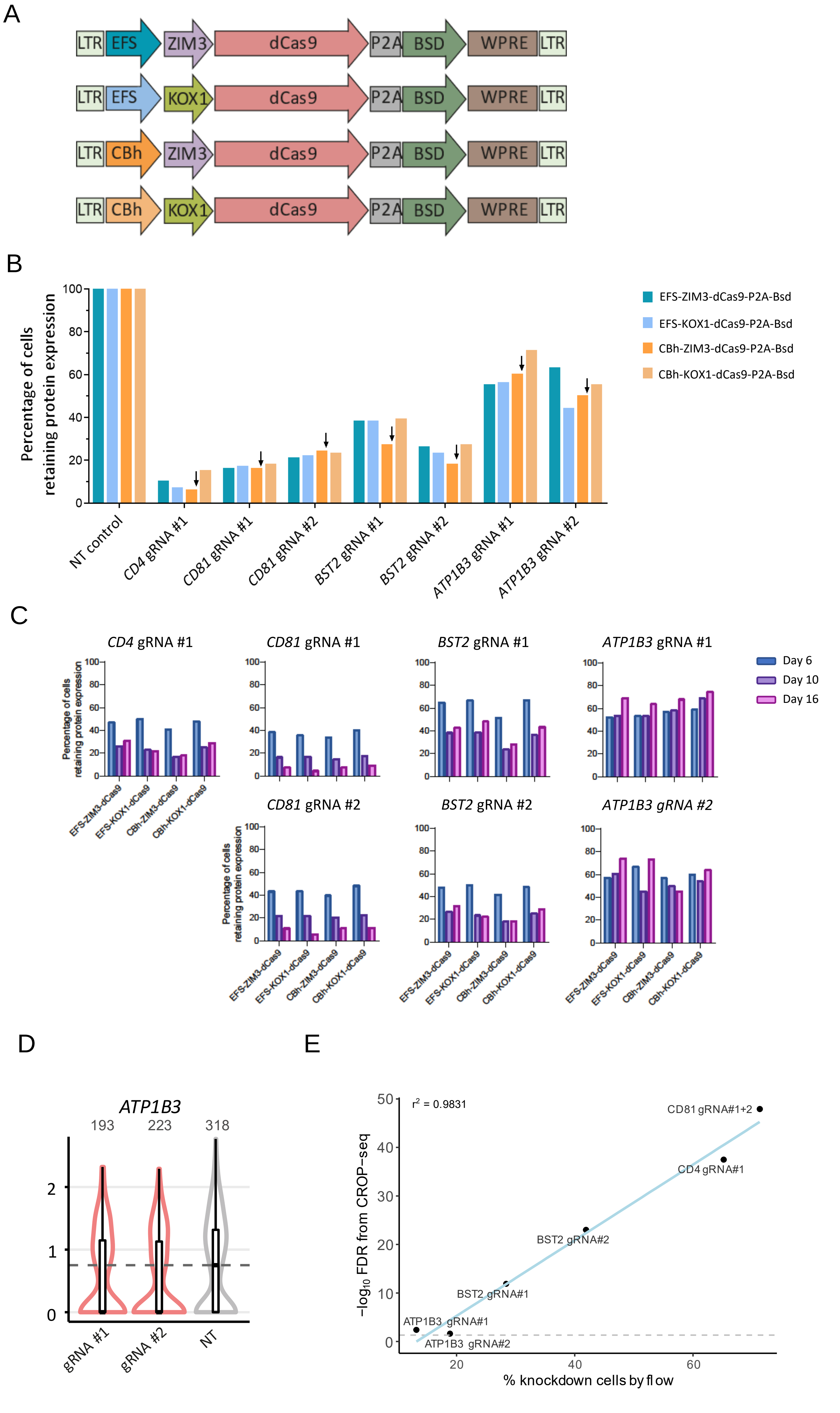

A
LTR EFS ZIM3 dCas9 P2A BSD WPRE LTR
LTR EFS KOX1 dCas9 P2A BSD WPRE LTR
LTR CBh ZIM3 dCas9 P2A BSD WPRE LTR
LTR CBh KOX1 dCas9 P2A BSD WPRE LTR
B
Percentage of cells retaining protein expression
EFS-ZIM3-dCas9-P2A-Bsd
EFS-KOX1-dCas9-P2A-Bsd
CBh-ZIM3-dCas9-P2A-Bsd
CBh-KOX1-dCas9-P2A-Bsd
NT control
CD4 gRNA #1
CD81 gRNA #1
CD81 gRNA #2
BST2 gRNA #1
BST2 gRNA #2
ATP1B3 gRNA #1
ATP1B3 gRNA #2
C
CD4 gRNA #1
CD81 gRNA #1
BST2 gRNA #1
ATP1B3 gRNA #1
CD81 gRNA #2
BST2 gRNA #2
ATP1B3 gRNA #2
Day 6
Day 10
Day 16
Percentage of cells retaining protein expression
EFS-ZIM3-dCas9
EFS-KOX1-dCas9
CBh-ZIM3-dCas9
CBh-KOX1-dCas9
D
ATP1B3
193
223
318
gRNA #1
gRNA #2
NT
E
r² = 0.9831
CD81 gRNA#1+2
CD4 gRNA#1
BST2 gRNA#2
BST2 gRNA#1
ATP1B3 gRNA#1
ATP1B3 gRNA#2
−log10 FDR from CROP−seq
% knockdown cells by flow

Fig. S1: A) Schematic of dCas9-KRAB lentiviral constructs tested. **B)** Bar plot showing the percentage of cells retaining protein expression for different target genes (*CD4, CD81, BST2, ATP1B3*) 10 days after TSS-targeting gRNA transduction into primary CD4$^+$ T cells expressing different dCas9-repressor constructs, analysed by flow cytometry and normalised to the corresponding non-targeting gRNA control sample. gRNA #1 and #2 refer to two different gRNA designs for a given TSS. **C)** Bar plots for the same experiment as B), showing flow cytometry data for days 6, 10 and 16 post-gRNA transduction. **D)** Normalised expression levels of *ATP1B3*, measured by 10X Genomics 3' scRNA-seq 11 days after the corresponding targeting (red) or non-targeting (grey) gRNAs were transduced into primary CD4$^+$ T cells expressing a CBh-ZIM3-dCas9 repressor construct. Dashed line indicates median expression level in cells with non-targeting controls. **E)** Percentage of cells showing protein downregulation of the target gene after CRISPRi by flow cytometry (x-axis) versus significance (-log10 FDR) of downregulation of the target gene (mRNA) by 3' scRNA-seq analysis (y-axis), normalised to the corresponding NT control. Pearson $r^2 = 0.98$.

Figure S2

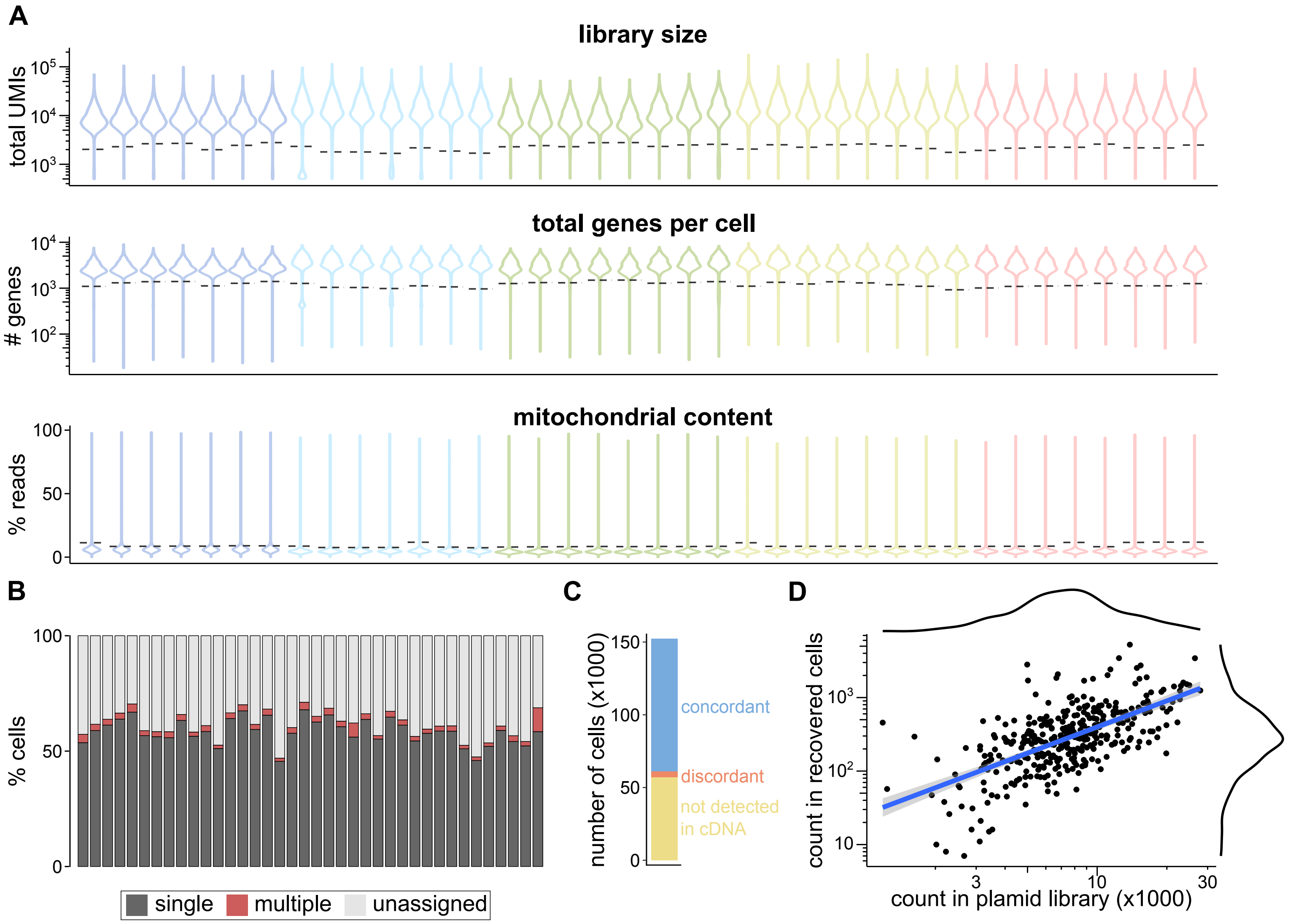
A
library size
total UMIs
10^5
10^4
10^3
total genes per cell
genes
10^4
10^3
10^2
mitochondrial content
% reads
100
50
0
B
% cells
100
50
0
single
multiple
unassigned
C
number of cells (x1000)
150
100
50
0
concordant
discordant
not detected in cDNA
D
count in recovered cells
10^3
10^2
10
3
10
30
count in plamid library (x1000)

Fig. S2: A) Distributions of the total UMIs per cell, number of detected genes per cell, and fraction of reads mapping to mitochondrial genes, used for quality control of the scRNA-seq data. Each violin corresponds to a technical replicate (channel in a 10X chip); colours indicate different 10X chips. The dotted lines indicate the thresholds used for each replicate to exclude poor-quality cells. **B)** Same as Fig. 2C but split per technical replicate. **C)** Barplot showing the number of cells where the same gRNA is the most abundant in both the cDNA and gRNA libraries (concordant, blue); the most abundant gRNA is different between libraries (discordant, orange); or no gRNA information was recovered from the cDNA library (yellow) **D)** Scatter plot of the relative abundance of each gRNA in the plasmid library (*x-axis*, assessed by DNA sequencing of the library) versus the number of cells positive for each gRNA (*y-axis*, assessed from the scRNA-seq data).

Figure S3

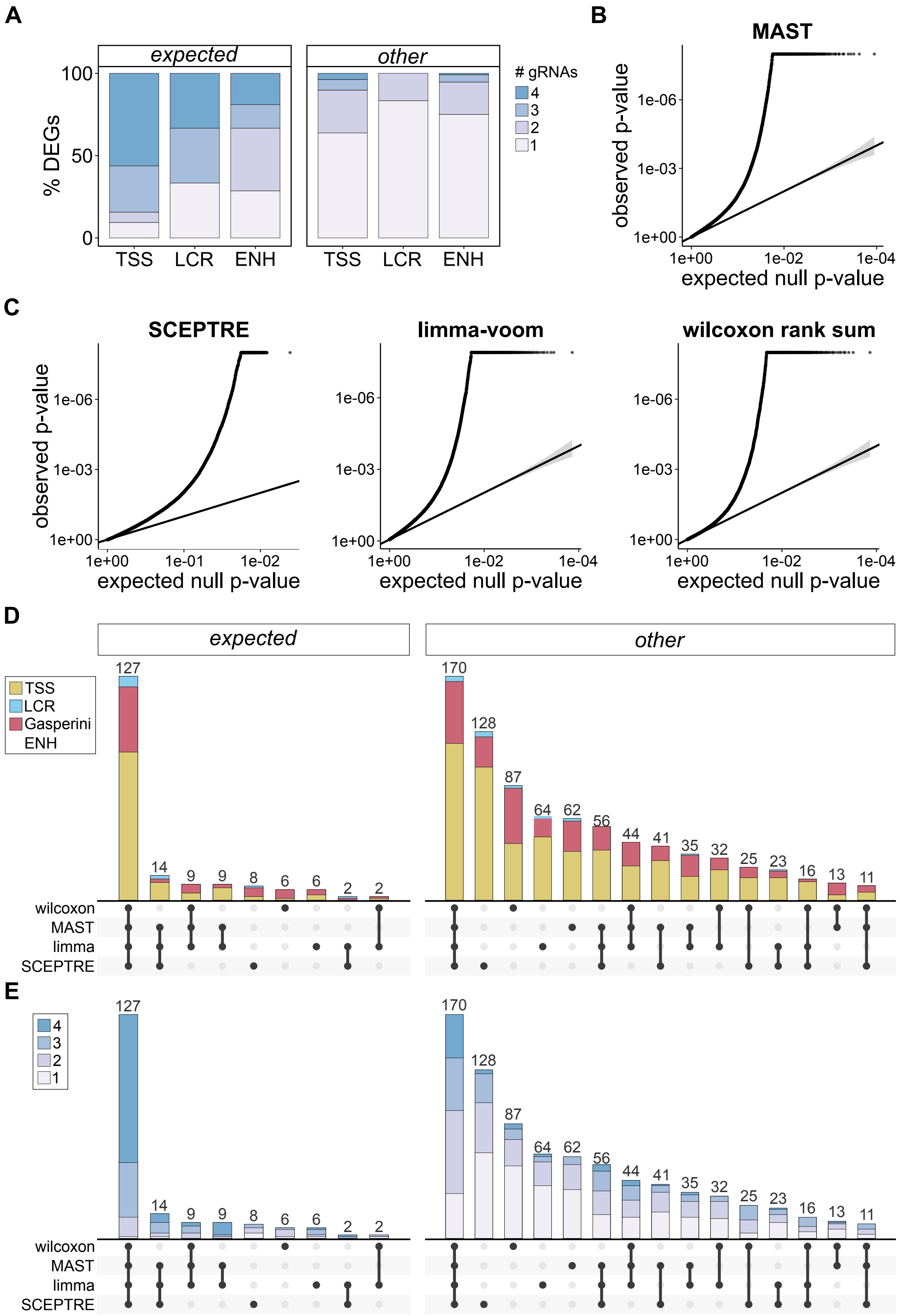
A
expected
other
% DEGs
100
50
0
TSS
LCR
ENH
gRNAs
4
3
2
1
B
MAST
observed p-value
expected null p-value
1e-06
1e-03
1e+00
1e-02
1e-04
C
SCEPTRE
limma-voom
wilcoxon rank sum
1e-01
D
TSS
LCR
Gasperini
ENH
127
14
9
8
6
2
170
128
87
64
62
56
44
41
35
32
25
23
16
13
11
wilcoxon
MAST
limma
SCEPTRE
E

Fig. S3: A) Barplots of the fraction of significant differentially expressed genes (DEGs) from positive control perturbations that are supported by different numbers of gRNAs (raw gRNA-level p-value < 0.05). The *expected* genes are shown separately from all *other* DEGs. **B)** QQ plot of the expected vs observed p-values reported by MAST when testing for expression changes from non-targeting gRNAs, which should not induce any significant changes in expression. Deviation from the diagonal indicates inflated p-values. **C)** Same as B) but for results from SCEPTRE, limma-voom or a Wilcoxon rank sum test. **D)** Upset plot indicating the number of DEGs for positive control targets that are identified by any of the four methods. The height of the bar indicates the number of DEGs, split by target class (indicated by different colours). Under each bar, the methods that called the gene as significant are indicated. The *expected* genes are shown separately from all *other* DEGs. **E)** Same as D) but with the colours indicating the number of gRNAs supporting each DEG (raw gRNA-level p-value < 0.05).
